# Supplementary material for: tRNA-Derived Fragments as Biomarkers in Bladder Cancer
Source: Cancers (Basel). 2024 Apr 20;16(8):1588. doi: 10.3390/cancers16081588 (PMC11049458; doi:10.3390/cancers16081588)
Supplement: Supplementary file 1 [file cancers-16-01588-s001.zip › cancers-2964846-supplementary.pdf]

Supplementary

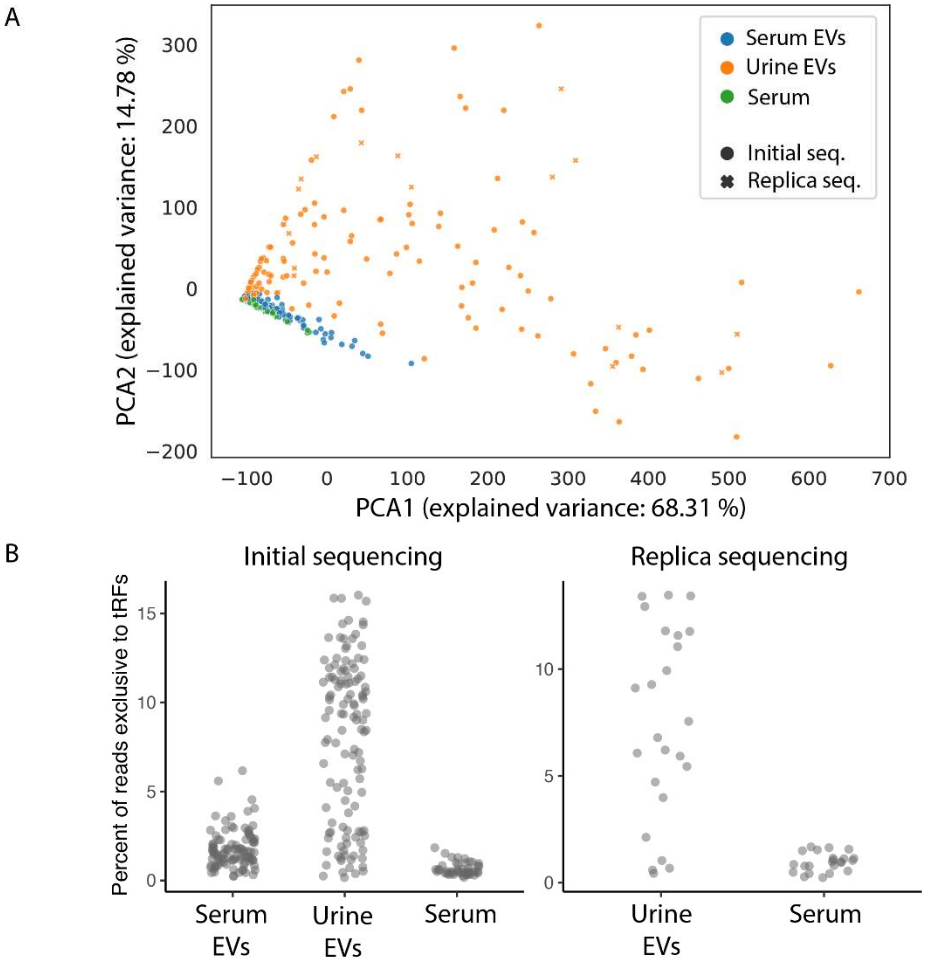

Figure S1. PCA plot and percentage of reads exclusive to tRFs. A) PCA plot of tRF counts from MINTmap using the exclusive tRF counts. B) Scatter plot made by counting total reads in exclusive tRF counts from MINTMAP, dividing it by total reads per sample and multiplying by 100.

FORESPØRSEL OM DELTAKELSE I FORSKNINGSPROSJEKTET

URO-ONKOLGISK FORSKNING OM EXOSOMER HOS URINVEISKREFTPASIENTER

Dette er et spørsmål til deg om å delta i et forskningsprosjekt for å se om man kan finne biologiske komponenter i urin som kan forbedre diagnostikken av kreft i urinveiene. Du får forespørsel om å delta i studien fordi du har blitt diagnostisert med kreft. I alle kroppsvæsker finnes det små partikler som kalles exosomer. Disse partiklene kan beskrives som små informasjonspakker som kan utveksles mellom celler. Det er kjent at det biologiske innholdet i exosomene endres ved kreftsykdom. Studien går ut på å analysere det biologiske innholdet i exosomene og se om dette endres etter behandling. Studien gjennomføres ved Avdeling for urologisk kirurgi, St. Olavs Hospital

HVA INNEBÆRER PROSJEKTET?

Prosjektet går ut på å samle inn urin og blod før og etter kirurgisk behandling av blærekreftpasienter. Dersom du velger å delta i studien, leverer du urinprøve og det blir tatt blodprøve på operasjonsdagen samt etter operasjonen. Du vil følge vanlig behandling og oppfølging. Urin og blod prøver vil bli tatt ved alle etterfølgende kontroller ca. 2 til 3 gange årlig. Blodprøver vil bli tatt ved vår avdeling. Urinprøveglass får du ved avdelingen.

I prosjektet vil vi innhente og registrere opplysninger om diagnose og behandling fra din pasientjournal.

MULIGE FORDELER OG ULEMPER

Det anses ikke å være noen fordeler eller ulemper ved deltakelse i studien. Behandlingsforløp og oppfølging påvirkes ikke av om du velger å delta i studien. Dere gjørs oppmerksom på at prosjektet ikke innebærer noe ekstra oppmøte ved St.Olavs Hospital ut over det som ellers gjøres i forbindelse med Deres behandlinger og kontroller.

FRIVILLIG DELTAKELSE OG MULIGHET FOR Å TREKKE SITT SAMTYKKE

Det er frivillig å delta i prosjektet. Dersom du ønsker å delta, undertegner du samtykkeerklæringen på siste side. Du kan når som helst og uten å oppgi noen grunn trekke ditt samtykke. Dette vil ikke få konsekvenser for din videre behandling. Dersom du trekker deg fra prosjektet, kan du kreve å få slettet innsamlede prøver og opplysninger, med mindre opplysningene allerede er inngått i analyser eller brukt i vitenskapelige publikasjoner. Dersom du senere ønsker å trekke deg eller har spørsmål til prosjektet, kan du kontakte: Carl-Jørgen Arum, tlf. 72571148, e-post: Carl-Jorgen.Arum@Stolav.no

HVA SKJER MED INFORMASJONEN OM DEG?

Informasjonen som registreres om deg skal kun brukes slik som beskrevet i hensikten med studien. Du har rett til innsyn i hvilke opplysninger som er registrert om deg og rett til å få korrigert eventuelle feil i de opplysningene som er registrert.

Alle opplysningene vil bli behandlet uten navn og fødselsnummer eller andre direkte gjenkjennende opplysninger. En kode knytter deg til dine opplysninger gjennom en navneliste. Prosjektleder har ansvar for den daglige driften av forskningsprosjektet og at opplysninger om deg blir behandlet på en sikker måte. Informasjon om deg vil bli anonymisert eller slettet senest fem år etter prosjektslutt.

I regi av Helsedirektoratet er der under etablering en Norsk Urotelkreft register hvor anonymiserte data fra alle Norske urotelkreft pasienter vil bli inkludert. I tillegg vil prosjektet benytte seg av sentrale norske helseregister inklusive Norsk Pasientregister, Norsk Kreftregister, Dødsårsaksregisteret, Sykehusinfeksjonsregisteret og Nasjonalt Blærekreftregister (under etablering).

HVA SKJER MED PRØVER SOM BLIR TATT AV DEG?

Urinprøvene og blod prøverne du leverer inn skal oppbevares i Biobank-1, St. Olavs Hospital (forskningsbiobanken) ved avdelingssjef Haakon Skogseth.

FORSIKRING

Norsk pasientskadeforsikring gjelder ved deltagelse i studien.

UTLEVERING AV OPPLYSNINGER TIL ANDRE

Datakontroll kan bli aktuelt og formålet er å kontrollere om studieopplysningene stemmer overens med tilsvarende opplysninger i din journal. Alle som får innsyn har taushetsplikt. Av kontrollhensyn blir grunnlagsdata oppbevart forsvarlig i 5 år etter avslutning av studien iht. helseforskningslovens §38. Deretter vil data bli slettet. Det er Dr. Carl-Jørgen Arum, overlege ved urologisk avd. St. Olavs Hospital, som er ansvarlig for datamaterialet i denne periode. Instanser som kan tenkes å kontrollere grunnlagsmaterialet er f. eks. forskningsansvarlige, uredelighetsutvalget for forskning og helsetilsynet.

OPPFØLGINGSPROSJEKT

Deltakelse i studien innebærer samtykke til å bli kontaktet i forbindelse med oppfølgingsprosjekt innen 2 år

ØKONOMI [TAS **KUN** MED HVIS DET ER AKTUELT]

Prosjektet har fått driftstøtte fra St. Olavs Hospital for gjennomføring av studien, det er ingen interessekonflikter knyttet til dette.

GODKJENNING

Prosjektet er godkjent av Regional komite for medisinsk og helsefaglig forskningsetikk, REK 2015/841/REK midt.

SAMTYKKE TIL DELTAKELSE I PROSJEKTET

JEG ER VILLIG TIL Å DELTA I PROSJEKTET

Sted og dato

Deltakers signatur

Deltakers navn med trykte bokstaver

Jeg bekrefter å ha gitt informasjon om prosjektet

Sted og dato

Signatur

Rolle i prosjektet
